# Vector delivery technique affects gene transfer in the cornea in vivo

**Published:** 2010-11-27

**Authors:** Rajiv R. Mohan, Ajay Sharma, Tyler C. Cebulko, Ashish Tandon

**Affiliations:** 1 Mason Eye Institute, School of Medicine, University of Missouri, Columbia, MO; 2 Ophthalmology Research, Harry S. Truman Memorial Veterans’ Hospital, Columbia, MO; 3 Department of Ophthalmology, College of Veterinary Medicine, University of Missouri, Columbia, MO

## Abstract

**Purpose:**

This study tested whether controlled drying of the cornea increases vector absorption in mouse and rabbit corneas in vivo and human cornea ex vivo, and studied the effects of corneal drying on gene transfer, structure and inflammatory reaction in the mouse cornea in vivo.

**Methods:**

Female C57 black mice and New Zealand White rabbits were used for in vivo studies. Donor human corneas were used for ex vivo experiments. A hair dryer was used for drying the corneas after removing corneal epithelium by gentle scraping. The corneas received no, once, twice, thrice, or five times warm air for 10 s with a 5 s interval after each 10 s hair dryer application. Thereafter, balanced salt solution (BSS) was topically applied immediately on the cornea for 2 min using a custom-cloning cylinder. The absorbed BSS was quantified using Hamilton microsyringes. The adeno-associated virus 8 (AAV8) vector (1.1×10^8^ genomic copies/µl) expressing marker gene was used to study the effect of corneal drying on gene transfer. Animals were sacrificed on day 14 and gene expression was analyzed using commercial staining kit. Morphological changes and infiltration of inflammatory cells were examined with H & E staining and immunocytochemistry.

**Results:**

Mice, rabbit or human corneas subjected to no or 10 s drying showed 6%–8% BSS absorption whereas 20, 30, or 50 s corneal drying showed significantly high 14%–19% (p<0.001), 21%–22% (p<0.001), and 25%–27% (p<0.001) BSS absorption, respectively. The AAV8 application on mouse cornea after 50 s drying showed significantly higher transgene delivery (p<0.05) in vivo with mild-to-moderate changes in corneal morphology. The 30 s of drying also showed significantly (p<0.05) high transgene delivery in mouse stroma in vivo without jeopardizing corneal morphology whereas 10 or 20 s drying showed moderate degree of gene transfer with no altered corneal morphology. Corneas that underwent 50 s drying showed high CD11b-positive cells (p<0.01) compared to control corneas whereas 20 or 30 s air-dried corneas showed insignificant CD11b-positive cells compared to control corneas.

**Conclusions:**

Controlled corneal drying with hair dryer increases vector absorption significantly. The dispensing of efficacious AAV serotype into cornea with optimized minimally invasive topical application technique could provide high and targeted expression of therapeutic genes in the stroma in vivo without causing significant side effects.

## Introduction

Gene therapy is an attractive approach to treat ocular diseases. Preclinical corneal gene therapy studies in animal models have shown promising results [1-5]. Nonetheless, untargeted and uncontrolled gene delivery remains a major challenge. The clinical application of gene therapy for corneal diseases is contingent on delivery of the therapeutic genes in a tissue selective manner. Our central hypothesis is that vector and vector-delivery techniques regulate transgene delivery in the cornea, and a suitable combination of efficacious vector and vector-delivery technique can successfully deliver therapeutic genes into keratocytes or stroma of the cornea in vivo. Among various gene transfer vectors tested for corneal gene therapy, viral vectors have emerged as favored vectors as they exhibited high transgene expression for longer duration [5-7]. We found adeno-associated virus (AAV) vectors to be highly efficient and safe for delivering foreign genes in rodent, rabbit, equine and human cornea in vitro and in vivo [5,6,8-11]. A varied degree of tissue selective tropism among AAV serotypes has also been observed for the cornea like other tissues [9-12]. The AAV serotypes 5, 8, and 9 showed superior transduction efficiency for transporting genes in the rodent or rabbit cornea in vivo among various tested AAV serotypes [10,11,13-15]. A careful literature survey revealed that the efficacy of numerous vectors has been examined for corneal gene therapy but no efforts have been made to study the role of vector-delivery techniques in corneal gene transfer.

The stroma constitutes 90% of the corneal tissue and its cellular components play important role in maintaining corneal transparency, function and pathology. The stroma is affected in a variety of corneal disorders such as graft rejection, haze, neovascularization, herpes keratitis, fibrosis and scarring. Gene therapy treatments without any side effects to treat stromal corneal disorders require localized expression of therapeutic genes into keratocytes and/or the stroma. For this purpose, numerous recombinant viruses and lipids were tested administering variable volume, concentration, strength, duration and frequency of vector in the cornea via microinjection, topical, electroporation, ultrasound or gene gun [16]. Most of these studies are focused on identifying an optimal vector and dose for delivering genes in the cornea in vivo. To the best of our knowledge, no study harvested vector delivery-techniques as a tool and studied its role for enhancing and targeting gene delivery in the corneal stroma in vivo.

The hydration and porosity of the cornea regulate its transparency [17]. The cornea constantly absorbs fluid from the aqueous humor and limbal blood vessels and becomes hazy if not pumped out by the endothelium [18]. The cadaver human corneas are often hazy due to absorption of aqueous humor but their transparency can be restored by incubating tissue in warm and ventilated chamber at 31 °C [19]. Furthermore, the drying of the cornea with a hair dryer is a known conventional treatment for Fuchs’ dystrophy among patients in eye clinic [20]. These observations prompted us to hypothesize that controlled drying of the cornea with a defined technique will deplete water content and increase vector absorption applied topically immediately on the dried cornea without jeopardizing corneal function. The present study tested this hypothesis using hair dryer, AAV8 vector and topical application technique. Topical application is the most acceptable method for delivering therapeutics in the eye. The specific aims of the study were to evaluate whether controlled drying of the cornea increases vector absorption in mouse and rabbit corneas in vivo and human cornea ex vivo, and examined the effects of defined drying technique on gene transfer, corneal structure and inflammatory reaction using an in vivo mouse model.

## Methods

### In vivo and ex vivo model

Six to eight week old female C57 mice (18–21 g) and New Zealand White rabbits (2.5–3.0 kg) were used for in vivo studies. The donor human corneas procured from eye banks were used for ex vivo investigations. All animals and human corneas were treated in accordance with the tenets of the ARVO Statement for the Use of Animals in Ophthalmic and Vision Research and the declaration of Helsinki. Mice were anesthetized with intramuscular injection of ketamine (130 mg/kg) and xylazine (8.8 mg/kg) whereas rabbits were anesthetized by intramuscular injection of ketamine hydrochloride (50 mg/kg) and xylazine hydrochloride (10 mg/kg). Topical ophthalmic 1% proparacaine hydrochloride solution (Alcon, Ft. Worth, TX) was instilled in each eye for local anesthesia.

### Topical drying and vector delivery technique

The corneal epithelium of the mouse and rabbit corneas was removed by gentle scraping with a #64 Beaver blade (Becton–Dickinson, Franklin Lakes, NJ) under an operating microscope under general and local anesthesia. The epithelium of human cornea was removed similarly after placing tissue on the dried surface of the culture dish. After removing corneal epithelium eyes were washed with BSS (Alcon) and wiped with a merocel sponge. The Conair hair dryer of 234Watts (Model 1875; Conair, Stamford, CT) was used for drying the rodent, rabbit, and human corneas. The temperature and air-flow of warm air were 41 °C and 6.8 m/s, respectively, according to digital Velocicheck anemometer (Model 8330; TSI Inc., Shoreview, MN). The hair dryer was operated from a distance of 8 inches and approximately 45° angle to the eye. The corneas either received no warm air (control) or warm air once for 10 s, twice for 10 s with 5 s interval, or thrice for 10 s with 5 s interval after every round or five times for 10 s with 5 s interval after every round. Immediately after drying, 2 µl BSS or vector was topically applied on the mouse cornea and 50 µl on the human and rabbit corneas for two min using a custom-cloning cylinder. The cloning cylinder of 3 mm diameter was used for the mouse and 7 mm diameter for the rabbit and human corneas.

### Quantification of vector absorption

Hamilton microsyringes (Hamilton, Reno, NV) and 0.2-2ul pipetman (Gilson, Middleton, WI) were used to dispense and quantify unabsorbed BSS or vector topically applied on the cornea using a cloning cylinder. After 2 min, all unabsorbed BSS/vector volume of the total 2 µl BSS/vector applied on the mouse cornea (n=12) or 50 µl BSS applied on the rabbit (n=12) and human (n=12) corneas was collected and measured. The amount of vector absorbed by the animal and human corneas was calculated by subtracting unabsorbed BSS/vector volume from the total applied BSS/vector volume. The results are expressed in percent.

### AAV vector production

AAV8 vector was generated using adenovirus free system following methods reported previously [10]. Briefly, human embryonic kidney (HEK) 293cells were co-transfected with AAV2-based genomic vector pARAP4, AAV8 Rep/Cap plasmid, and adenovirus helper plasmid in a ratio of 1:3:3. The pARAP4 expresses heat stable placental alkaline phosphatase (AP) under the regulation of Rous sarcoma virus (RSV) promoter/enhancer and simian virus 40 (SV40) polyadenylation sequence. The virus containing cell lysate was harvested at 62 h post-transfection. Recombinant viral stocks were purified by two sequential rounds of CsCl gradient ultracentrifugation. Collected viral fractions were pooled and dialyzed through two rounds of HEPES-buffered saline. Viral titer was determined by dot blot analysis using DIG labeled probes (Roche Applied Science, Indianapolis, IN). The AAV genomic plasmid (pARAP4) was obtained from Dr. Dusty Miller, Fred Hutchison Cancer Research Center, Seattle WA and pAAV2/8 plasmid was procured from Dr. James M. Wilson, Gene Therapy Program, Division of Medical Genetics, University of Pennsylvania, Philadelphia PA.

### AAV8 application to mouse cornea

Thirty-six female C57 mice were used to study the effects of drying the levels of gene transfer. The study was approved by the Animal Care and Use Committees of the University of Missouri-Columbia and Harry S. Truman Memorial Veterans’ Hospital Columbia, MO. Mice were given general anesthesia with intramuscular injection of ketamine and xylazine and local anesthesia by instilling 1% proparacaine hydrochloride on the eye. After removing epithelium, 2 µl BSS or AAV8 (1.1×10^8^ genomic copies/µl) was topically applied on mouse cornea for 2 min using a custom-cloning cylinder (3 mm in diameter) as described earlier under vector delivery technique. The mice were divided into 5 groups: Group-1 corneas received warm air for 10 s, Group-2 corneas received 2 rounds of warm air for 10 s with 5 s interval, Group-3 received 3 rounds of warm air for 10 s with 5 s interval after every round, Group-4 received 5 rounds of warm air for 10 s with 5 s interval after every round and Group-5 corneas did not receive any warm air after merocel wiping and served as a control. All animals were sacrificed at 14 days after BSS/vector application.

### Tissue embedding

Mouse eyes were enucleated and embedded in liquid OCT compound (Sakura FineTek, Torrance, CA) within a 15 mm × 15 mm × 5 mm mold (Fisher Scientific, Pittsburgh, PA) and snap frozen as reported previously [10]. The frozen tissue blocks were maintained at −80 °C. Tissue sections (7 μ thick) were cut with a cryostat (HM 525M; Microm GmbH, Walldorf, Germany) and maintained frozen at −80 °C until staining.

### Tissue morphology and gene delivery

Corneal tissue morphology was analyzed with hematoxylin and eosin (Fisher Scientific) staining following vendor’s protocol. The delivered marker alkaline phosphatase (AP) gene expression was determined with cytochemical staining following manufacturer’s instructions. In brief, tissue sections were washed with HEPES buffer and incubated with a mixture of 5-bromo-4-chloro-3′-indolylphosphate p-toluidine (BCIP) and nitro-blue tetrazolium (NBT) at 37 °C. The AP-stained corneal stroma appeared dark blue. Gene transfer was quantified by determining the pixels of AP stained area in 400× magnification using National Institutes of Health Image J 1.38X (NIH, Bethesda, MD) software.

### Inflammatory response

The effects of corneal drying on inflammatory reaction were analyzed by the CD11b and F4/80 immunocytochemistry. The immunofluorescence staining for CD11b (BD PharMingen, San Jose, CA) and F4/80 (Serotec, Raleigh, NC) was performed using rat anti-mouse antibodies. Tissue sections (7 µm) were washed with 1× HEPES, blocked in 5% BSA for 30 min followed by incubation at room temperature with the primary antibody at 1:50 dilution for 90 min and with secondary antibody goat anti-rat IgG (AlexaFlour 594; Molecular Probes, Eugene, OR) at a dilution of 1:500 for 60 min. Vectashield mounting medium containing DAPI (Vector Laboratories, Inc. Burlingame, CA) was used to visualize nuclei in the tissue sections. The sections were viewed and photographed under a Leica fluorescent microscope (Leica, Wetzlar, Germany) equipped with a digital camera.

### Statistical analysis

The results were expressed as mean±standard error of the mean (SEM). Statistical analysis was performed using either one-way ANOVA (ANOVA) followed by Tukey’s multiple comparison test or two-way ANOVA followed by Bonferroni test.

## Results

### Topical vector delivery technique

**Figure 1:**
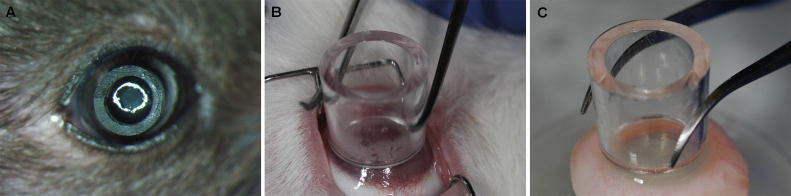
Representative image showing newly-defined vector application technique using cloning cylinder. **A**: Mouse cornea, in vivo. **B**: Rabbit cornea, in vivo. **C**: Human cornea, ex vivo.

Figure 1 shows details of the defined vector application technique developed for controlled and targeted gene delivery in the stroma, in vivo. As evident from figure, dispensing of BSS/vector inside a cloning cylinder limits its contact to neighboring ocular tissue. This significantly enhanced transgene delivery into the targeted corneal stroma and prevented transgene delivery into untargeted tissues such as limbus, conjunctiva, sclera, etc.

### Effect of corneal drying on absorption of topically applied solution

**Figure 2:**
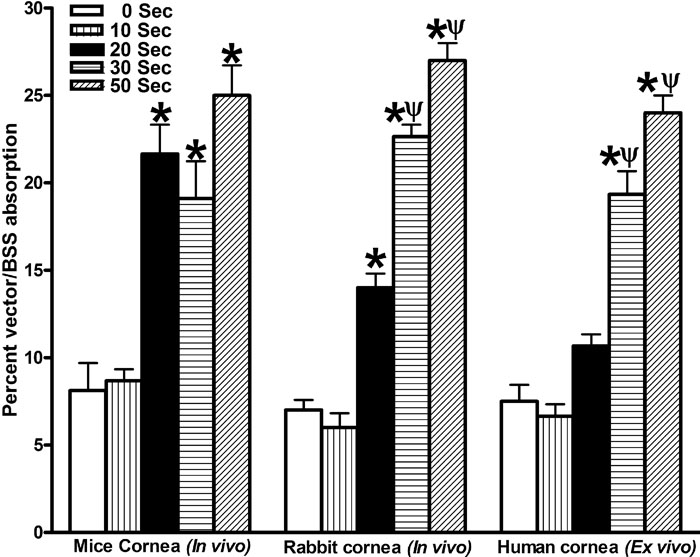
Effect of corneal drying on vector absorption in mice, rabbit and human corneas. Corneas were subjected to zero (0 s), one (10 s), two (20 s), three (30 s), or five (50 s) rounds of warm air drying and 2 µl (mouse) or 50 µl (rabbit and human) of vector/BSS was applied onto corneas for 2 min immediately after drying. The amount of vector absorbed by the corneas was calculated by subtracting unabsorbed BSS/vector volume from the total applied BSS/vector volume. The results are expressed in percent as mean±SEM *p<0.001 as compared to 0 s or 10 s; ψp<0.001 as compared to 20 s.

Figure 2 shows the effect of corneal drying on absorption of topically applied BSS solution in mouse, rabbit, and human corneas. Mice corneas subjected to 0 or 10 s drying showed 8±1.5% absorption of topically applied BSS solution. Drying for 20, 30, or 50 s significantly (p<0.001) enhanced the absorption to 21±1.4%, 19±2.1%, and 25±1.5%, respectively. No statistical significance in corneal absorption was noted between 20, 30, or 50 s of drying, which may be because of small volume (2 µl) of topically applied solution.

Zero and 10 s drying of rabbit corneas showed 7±1.1% absorption of topically applied BSS solution. Twenty seconds of warm air drying significantly (p<0.001) enhanced corneal absorption to 14±0.8%. Thirty and 50 s of warm air drying in rabbits resulted in further increase in corneal absorption to 22±0.6% and 27±0.8% (p<0.001; Figure 2).

Human corneas exposed to 0 or 10 s of drying showed 7±0.6% absorption of topically applied BSS solution. Twenty seconds of warm air drying enhanced corneal absorption to 10±0.6% but the increase was not statistically significant. Corneas subjected to 30 or 50 s of drying showed 19±1.15% and 24±0.9% absorption which was significantly (p<0.001) more compared to corneas subjected to either zero or twenty cycles of air drying (Figure 2).

### Effect of corneal drying on gene expression

**Figure 3:**
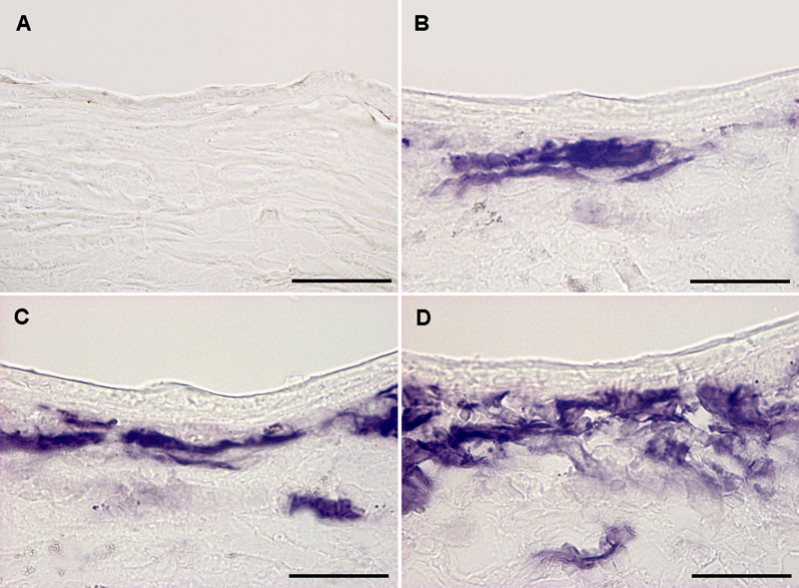
Representative images showing alkaline phosphatase marker gene delivery (purple) detected on day 14 in control (naive) and AAV-treated mouse cornea in vivo that received warm air. **A**: Naive. **B**: 20 s. **C**: 30 s. **D**: 50 s. Scale bar denotes 50 µm.

We next tested the effect of different rounds of corneal drying on AAV-mediated gene expression. Figure 3 shows AAV8-mediated gene expression in mice corneas subjected to 20, 30, or 50 s drying. Corneas exposed to 20 or fewer seconds of drying showed mild to moderate level of gene expression. As is evident from the figure, corneas exposed to 30 s of drying demonstrated significantly more gene expression. Fifty seconds of drying resulted in a further enhancement of gene expression (Figure 3).

**Figure 4:**
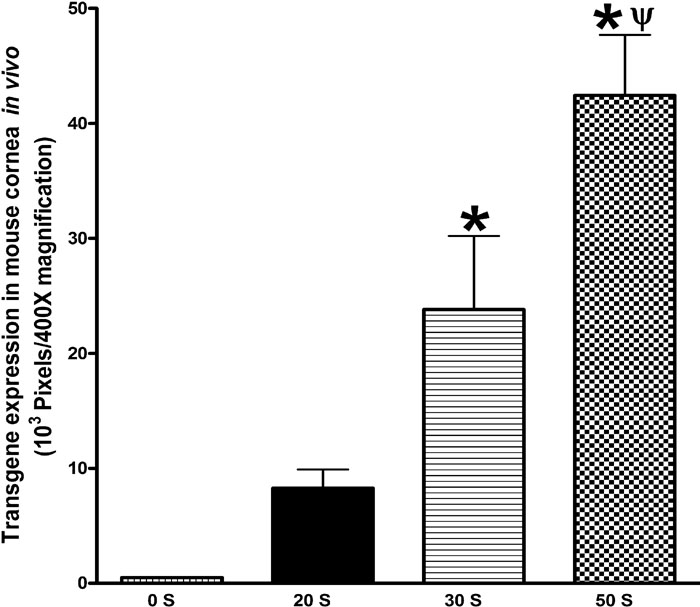
Digital quantification of delivered marker gene expression detected at day 14 in mouse corneas in vivo. These corneas received 2 µl of AAV8 vector immediately after 0, 20, 30, or 50 s of drying. The corneas that underwent 50 s of drying showed the highest level of gene expression followed by 30 s and then the 20 s group. *p<0.05 as compared to 20 s; ψp<0.05 as compared to 30 s.

Figure 4 shows the quantification of gene expression in corneas subjected to 20, 30, or 50 s of warm air drying. Corneas exposed to 50 s of drying showed the highest level of gene expression and it was significantly (p<0.05) more compared to groups subjected to 20 and 30 s of drying. Relative comparison between the 30 s and 20 s treatment group revealed significantly (p<0.05) higher levels of gene expression in corneas exposed to 30 s of drying.

### Effect of corneal drying on corneal morphology and inflammatory response

**Figure 5:**
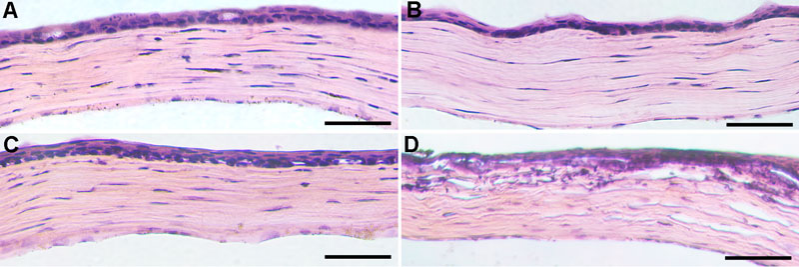
Representative images of H&E staining showing histology of mouse corneas subjected to air drying and collected on day 14 after vector application. **A**: Naive. **B**: 20 s. **C**: 30 s. **D**: 50 s. Corneas exposed to ≤30 s of drying showed normal morphology whereas corneas subjected to 50 s of drying showed moderate morphological changes in anterior stroma. Scale bar denotes 50 µm.

To test the possibility of corneal injury due to warm air drying, we performed histological examination by hematoxylin and eosin-staining of mice corneas. Figure 5 shows hematoxylin and eosin-stained corneal sections obtained from mice subjected to 0, 20, 30, or 50 s of warm air drying. No apparent structural abnormalities were detected in mice corneas subjected to 30 or fewer s of warm air drying. On the other hand, mice corneas exposed to 50 s of warm air drying showed structural damage in the anterior stroma. A large number of hematoxylin-stained nuclei were also detected in the anterior stroma of these corneas suggesting the possibility of inflammatory cells.

**Figure 6:**
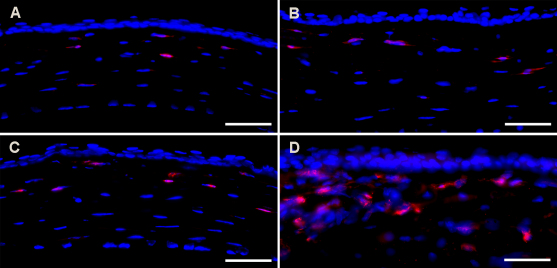
Representative immunohistochemistry images showing CD11b+ cells in naive and AAV-treated mouse corneas subjected to warm air and collected on day 14 after vector application. **A**: Naive. **B**: 20 s. **C**: 30 s. **D**: 50 s. DAPI stained nuclei are shown in blue and CD11b+ cells are shown in red. Scale bar denotes 50 µm.

The presence of inflammatory cells was confirmed by imunostaining for CD11b, a marker for activated granulocytes, and F4/80, a macrophage specific antigen. Figure 6 shows CD11b staining in corneas exposed to 0, 20, 30, or 50 s of warm air drying. Mice corneas subjected to ±30 s of warm air showed 7±2 CD11b+ cells and 50 s showed 33±5 CD11b+ cells, suggesting increase infiltration of activated granulocytes in severely dried cornea. The F4/80, marker for macrophages, showed similar results (data not shown).

## Discussion

Recent success in vision restoration in patients suffering with Leber congenital amaurosis with gene therapy affirms its promise to treat eye diseases [21,22]. Gene therapy approaches for corneal diseases is still in a developmental phase in which practical considerations for safety and efficacy have dominated the efforts. Numerous viral and non-viral vectors have been identified for this purpose [5-7]. Our laboratory recently studied the efficacy and toxicity of multiple AAV serotypes for delivering genes in the stroma using in vitro [9,11] and in vivo [10,13] models. To reduce side effects, improve safety and augment targeted delivery of genes into keratocytes or the stroma in vivo with AAV we are attempting to define vector-delivery techniques. It is our central hypothesis that vector and vector-delivery techniques regulate transgene delivery in the cornea in vivo. In this study, we report a simple minimally invasive hair-dryer based topical vector-delivery technique that significantly increases AAV-mediated transgene delivery in the mouse stroma in vivo, without compromising typical corneal morphology or function. Furthermore, we report that controlled drying of the mouse, rabbit and human corneas with hair dryer increases vector absorption in these tissues, and likely is the reason for increased transgene delivery in the mouse stroma in vivo.

Corneal hydration is regulated by epithelial barrier, proteoglycans’ water holding capacity, endothelial water-pumping mechanism, intraocular pressure and evaporation of fluid from the corneal surface and is critical for corneal function and clear vision [23,24]. In addition to these factors many agents such as benzalkonium chloride, cetylpyridinium chloride, EDTA, polyethoxylated castor oil, sodium deoxycholate etc present in ophthalmic topical formulations have been shown to influence corneal hydration [23,25]. We postulated that controlled drying of the cornea for short duration augments fluid absorbing capacity of the cornea until corneal hydration returns to the normal levels. Indeed, increased uptake of topical solution after corneal drying was observed in mouse, rabbit, and human corneas. Based on these observations, we conclude that enhanced transgene expression noted in mouse cornea in vivo after the topical application of AAV vector on de-epitheliazed air-dried corneas is because of increased vector absorption. The hairdryer-assisted corneal drying was chosen because this technique is an acceptable conventional treatment in clinical practice for corneal abnormalities such as Fuchs’ dystrophy [20]. We observed that application of 5 rounds of 10 s air drying compromised corneal morphology in vivo and 3 rounds of 10 s drying significantly augmented gene transfer without jeopardizing corneal morphology and 1 or 2 rounds of 10 s drying augmented only mild-to-moderate degree of gene transfer with no altered corneal morphology. These observations suggest that identification of duration of corneal drying for each species may be required.

Corneal epithelium acts as a strong barrier to topical gene delivery. We found that removal of corneal epithelium is necessary for achieving larger and targeted gene delivery into the stroma of mouse and rabbit corneas in vivo [8,10,13]. In the present study, the epithelium was removed via mechanical scraping as this technique is routinely used clinically in refractive laser surgical procedures such as photorefractive keratectomy, laser epithelial keratomileusis, etc. Mechanical removal of epithelium is known to induce keratocyte apoptosis, inflammation and wound healing in the cornea [26,27]. We [26,27] and other researchers [28] have shown that release of cytokines and growth factors following epithelial injury that ignite a transient wound healing response in the cornea. No additional inflammatory response was observed in the corneas subjected upto 3 rounds of warm air drying as supported by the detection of statistically comparable level of inflammatory cells in mouse corneas exposed to zero or 3 rounds of ten seconds drying. Significantly more inflammatory cells were noted in the corneal sections receiving 5 rounds of 10 s drying.

In conclusion, we demonstrate that controlled drying of the cornea with hair dryer is safe and results in increased vector absorption and consequently enhanced transgene delivery in the cornea. Furthermore, the newly optimized minimally invasive topical vector-delivery technique can be used in combination with AAV vector to achieve therapeutic levels of genes in the stroma of the cornea in vivo without causing significant side effects.
